# Mapping internal strain fields of fused filament fabrication metal filled polylactic acid structure using digital volume correlation

**DOI:** 10.1177/00219983231171658

**Published:** 2023-04-21

**Authors:** Abhinav Goyal, Cristofaro S Timpano, Garrett W Melenka

**Affiliations:** Department of Mechanical Engineering, 7991 York University, Toronto, ON, Canada

**Keywords:** Digital volume correlation, micro-computed tomography, fused filament fabrication, poly(lactic) acid

## Abstract

With the advancement in Fused Filament Fabrication (FFF), its application is increasing widely across different industries such as aeronautical, biomedical, robotics, etc. The internal structure is becoming more complex and intricate with varying materials of reinforcement which are used to improve mechanical properties. Current measurement techniques like Digital Image Correlation (DIC) are non-destructive testing methods that do not provide enough information on the behaviour of internal microstructure for anisotropic FFF materials. Digital Volume Correlation (DVC) is non-destructive testing technique which provides full field internal 3D deformation and strain fields. Copper particle filled PLA samples manufactured using FFF method with 20, 40, 60 and 80 infill percentages were loaded in tension inside Micro-CT. X-rays were passed through the sample to get a volumetric dataset for different loadings. Using DVC method on the dataset, internal displacement and strain fields were generated for 20, 40, 60 and 80 infill percentage FFF sample.

## Introduction

With the increasing use of fused filament fabrication (FFF) in various industries, FFF has become the most widely used 3D printing process.^1^ FFF is an additive manufacturing (AM) process where a filament is passed through a heated nozzle to form a solid body. Geometry is created from the layer-by-layer deposition of molten thermoplastic filament onto a build plate. FFF is widely used for part replacements, prototypes, moulds, and even load-bearing structures like in the field of aeronautics,^2^ construction^3^ and biomedicine.^4–6^ The materials commonly used are thermoplastic polymers like Polylactic Acid (PLA) or acrylonitrile butadiene styrene (ABS), both of which do not have high strength capacity. Various studies have been done to reinforce PLA or ABS with reinforced materials with fibres or metal particles to improve the strength further.^7^ Haijun He *et al.* fabricated a 3D printed nanocomposite using the FFF technique; both the filament and the nanofibres were manufactured with PLA, the latter through electrospinning.^8^ It was found that the tensile strength of 3d printed nanocomposite with 10.1% nanofibre content increased from 55.6 to 64.8 Mpa, an increase of 16.55%. Mohammadizadeh *et al.* studied on tensile strength of 3D printed fibre-reinforced nylon composites.^9^ And it was found that carbon fibre (CF) reinforced 3D with a volume fraction of 60% has a tensile strength of 446.87 MPa, an increase of 2231% from the strength of Nylon and greater than the strength of Aluminium 6061. These studies clearly show how introducing reinforcements improve the strength of FFF structures.

3D printed parts with reinforced material require an understanding of internal microstructure for which Micro-Computed Tomography (Micro-CT) is ideally suited. Micro-CT is a non-destructive method that uses X-rays to image an object’s internal microstructure.^10^ The X-ray beams are ejected through the X-ray tube towards the sample. The resulting image is based on the attenuation coefficient of the material. The attenuation coefficient is the measure of how easily beams are absorbed and transmitted with different intensities.^11^ The detectors are placed at the end, which collects the transmitted X-rays, which is the 2D projection of the sample. The sample is rotated 180 or 360 degrees to acquire numerous projections from different angles. After the projections are collected, an image reconstruction process gives a stack of cross-sectional images of the sample. S Sommacal *et al.* used Micro-CT to understand the voids in a 3D printed carbon-reinforced PEEK sample and the filament feedstock.^12^ The paper finds that the printing process had a minor effect in removing the voids present in the filaments. However, the depositional printing process greatly affected the internal void and fibre distribution microstructure. Yu *et al.* examined the microstructure of basalt fibre-reinforced PLA using Micro-CT.^13^ The result shows the effect of printing direction on the voids formation and microstructural anisotropy, which can lead to customized elastic modulus depending upon the printing direction.

AM parts have complex structures with different printing parameters. Printing parameters include raster orientation, infill percentage, and layer height. Due to these factors, the mechanical properties of AM parts are highly dependent on printing parameters.^14,15^ To understand the behaviour of printing parameters on loading conditions, various surface measurement techniques like Digital Image Correlation (DIC) have been used.^16,17^ DIC is a contact-free optical measurement technique to measure a specimen’s outer surface deformation and strain fields. However, DIC has limitations. For example, surface deformation behaviour does not correlate with the internal microstructure of the FFF part. H Gonabadi *et al.* studied the effect of build orientation, infill density, and pattern using DIC.^18^ They used a single camera to acquire images during tensile and shear testing. The authors found that 0° on-edge build orientation, a specimen manufactured on the edge of the sides with the plate support instead of a face, has better mechanical properties than other build orientations. But the mechanical behaviour for different infill patterns was similar. The increase in infill density leads to an increase in tensile properties in a quadratic manner. However, it does not depict the microscopic behaviour inside the sample, which is more prone to failure than the shell. Therefore, there is a need for full-field strain measurement, which correlates strain and deformation behaviour with the internal microstructure of FFF components.

Digital Volume Correlation (DVC) is an advanced measurement technique to measure the 3D deformation and strain of an object; DVC is similar to DIC but in a 3D space. DVC can use volumetric data such as X-ray CT or magnetic resonance imaging (MRI) data to measure deformation and strain.^19,20^ DVC was first developed by Bay *et al.* on a trabecular bone placed under compressed loading to study the internal strain.^21^ DVC was initially used for biomechanical applications and later extended to solid mechanics.^19^ Wang *et al.* used DVC for a porous 3D structure printed using stereolithography to find the deformation fields and relate the microstructure with the macrostructure.^22^ Timpano *et al.* studied the full-field internal displacement and the strain tensor of 100% infill density FFF sample with +45/−45° raster orientation.^23^ The result shows strains’ development along the printed sample’s internal raster. Despite initial work on the examination of FFF samples using the DVC method, further investigation using this technique is required.

The paper aims to study the effects of 20, 40, 60 and 80% infill percentages on a FFF part. This is achieved with the help of a Micro-CT machine and integrated testing stage to capture the cross-sectional images of a specimen under tensile loading of 50 N with a step loading of 150 N. After collecting Micro-CT image data, the DVC method will correlate different infill percentages to map full-field volumetric deformation and strain fields. The study will provide great insight into the complex structure of FFF, and the relation of microstructure with macrostructure behaviour can be established. Further, it can support validating Finite Element Analysis models for FFF specimens.

## Methodology

### 3D printing

**Figure 1.**
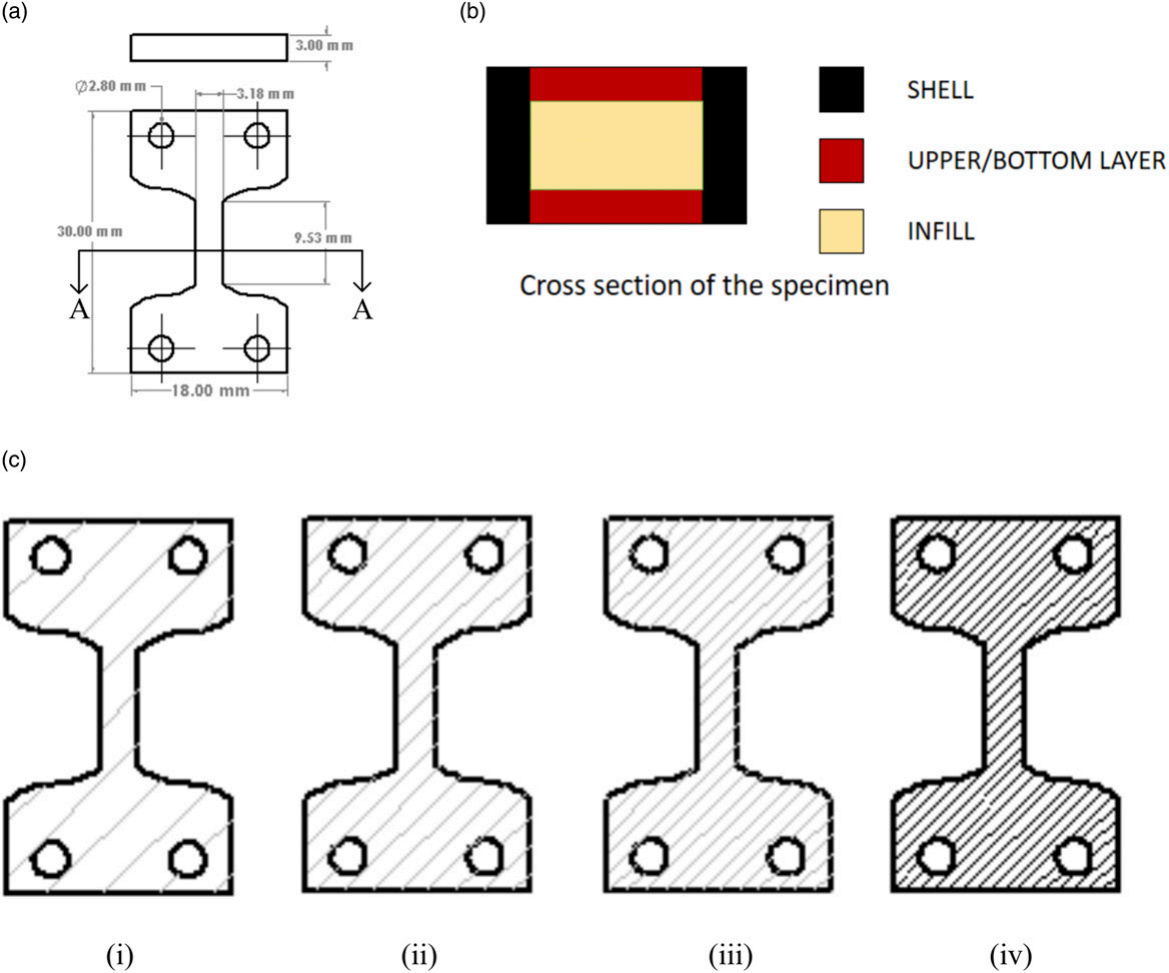
(a) Modified ASTM D638-14 type V Dimensions (b) shows the cross-section of geometry, which includes shell, upper/bottom layer and infill region (c) Infill pattern of (i) 20% (ii) 40% (iii) 60% (iv) 80%.

A modified ASTM D638-14 type V, as shown in Figure 1(a), was modelled for the given analysis. A solid model is generated using the given dimensions in CAD software (Solidworks 2020, Dassault Systèmes, France). Then the file is further imported into a 3D printing software (Prusa Slicer 2.2.0, Czech Republic) to define printing parameters like infill percentage and layer thickness described below in Table 1. Based on the parameters, specimens with 20, 40, 60 and 80 infill percentages are printed using an open-source 3D printer (Prusa i3 MK2, Czech Republic) by FFF. The filament used for printing is copper metal particle reinforced PLA (Metal filled PLA, CCTree-Mech Solutions LTD, Concord, Ontario). Figure 1(b) describes the cross-sectional view of the specimen and Figure 1(c) highlights the internal raster orientation between 20, 40, 60 and 80 infill percentages.

**Table 1.** 3D printing parameters.

Parameters	Copper filled PLA
Layer thickness	0.1 mm
Top/Bottom layer	0.8 mm
Shell thickness	0.4 mm
Infill %	20, 40, 60, 80
Support	Raft
Orientation	+45°/−45°
Nozzle temperature	200°C
Build plate temperature	60°C
Print speed	60 mm/s

### Microscopic image

**Figure 2.**
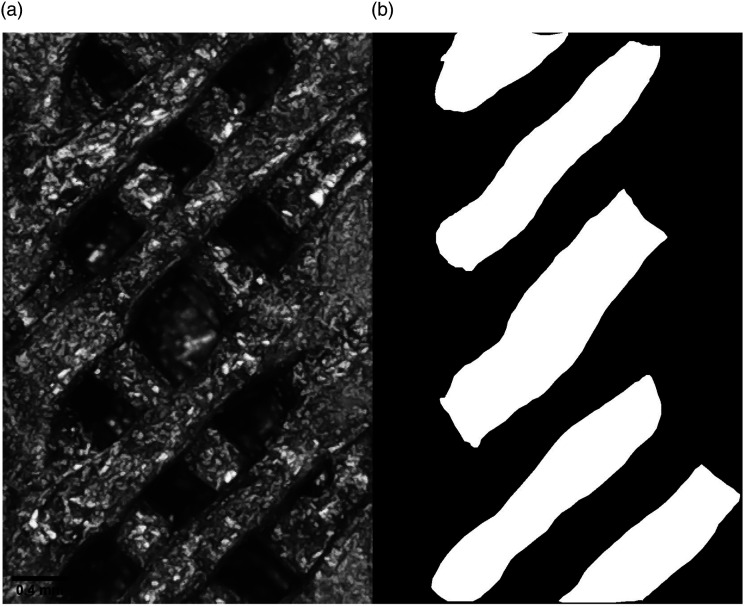
Steps for processing image (a) Captured microscopic image of 60% infill part (b) Final masked image for calculating porosity.

A magnified image of a 3D printed part section with infill patterns was captured using a microscope (Elikliv DM4, China). Cross-section images were captured to verify the Micro-CT results and to provide initial insights into the cross-section of the FFF components. The narrow section of the structure with a 60% grid infill pattern was captured, as shown in Figure 2(a). However, only a single image of the infill pattern was captured. The image was first converted into an 8-bit grayscale image, and using ImageJ (National Institutes of Health, Bethesda, Maryland, USA) ROI manager, the pore boundary was drawn manually. Further, using the overlay and mask in-built functions, the final image with pores in the white and solid region in black was generated, as shown in Figure 2(b). The porosity of the 2D image was calculated for a 60% grid infill pattern using image processing software CTAN (1.16, Bruker, Belgium).

### Micro-CT scans

X-ray Micro-CT was conducted using a desktop machine (Skyscan 1272 microCT, Bruker, Belgium). The specimen was mounted inside the Micro-CT machine on the material testing stage (Tension and Compression stage, Bruker, 440 N). Various parameters need to be set before the acquisition of a 3D image. Based on the specimen dimension and expected deformation, a pixel size of 5.4 μm has been selected with a resolution of 1468 × 1468 × 1561 pixels giving an effective field of view of 7.93 mm × 7.93 mm × 8.42 mm. A voltage of 70 KV is selected, and a 1 mm aluminum filter is applied based on recommended settings for the SkyScan 1272 machine. The added aluminum filter eliminates artifacts and noise from forming in the sample images. Frame averaging of 4 with 2 × 2 binning mode has been selected. During the acquisition of images from Micro-CT, multiple images can be captured at each rotation step, averaged out to give the final image, known as frame averaging, which leads to an increase in the signal-to-noise ratio. Binning is the process of combining pixels to form a larger pixel; for 2 × 2 binning mode, a matrix of 2 × 2 pixels is combined to create a single pixel which leads to an increase in signal-to-noise ratio. The rotation step corresponds to the increments in the sample that will be rotated for each scan. The rotation step affects the total scan time. Based on the recommendation by the manufacturer, a rotation step of 0.4° has been selected. A scan rotation of 360° is set, defining the sample’s total rotation throughout the acquisition. The scan settings for the Micro-CT are summarized in Table 2.

**Table 2.** Micro-CT scan parameters.

Micro-CT parameter	Setting
Source voltage	70 kV
Resolution	1468 px × 1468 px × 1561 px
Pixel size	5.4 μm
Filter	1 mm Al
Binning	2 × 2
Frame averaging	4
Rotation step	0.4°
Distance between X-ray source and sample	81.69 mm

**Figure 3.**
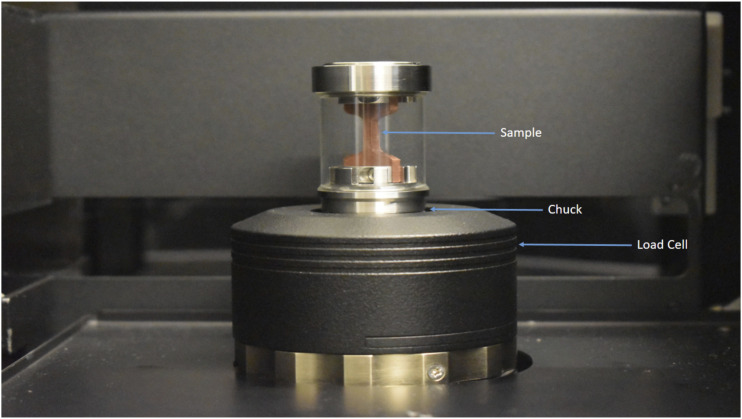
Setup view of the material testing stage (MTS) inside the Micro-CT.

Material Testing Stage (440 N integrated test stage, MTS2, Bruker, Belgium) parameters are adjusted after setting up the specification of scans. First, the load cell platen is fully extended. The sample is loaded into the sample holder, and then the sample holder is placed over the chuck in MTS, as shown in Figure 3. The MTS is operated in tensile mode for the in-situ test, and the load is increased slightly. Batch scanning is done to obtain multiple scans of the same object during deformation. A preload of 50 N was applied so that the sample remained rigid, and a step load of 150 N was applied. A loading rate of 4 mm/s was used to apply load to the test samples, and a delay of 180 s was used to ensure consistent results between loading steps.

**Figure 4.**
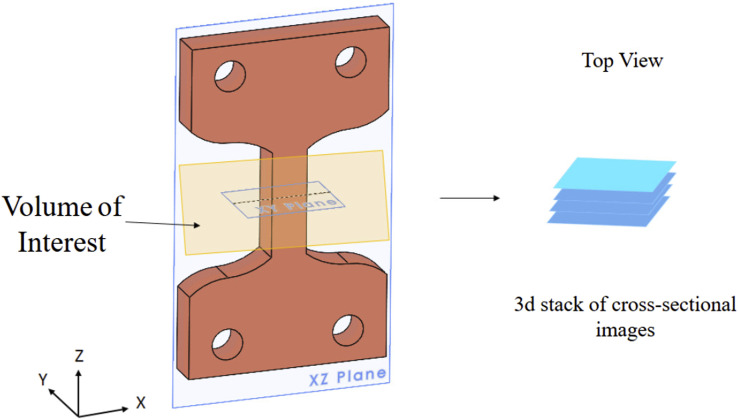
Schematic of Image reconstruction from Micro-CT.

**Figure 5.**
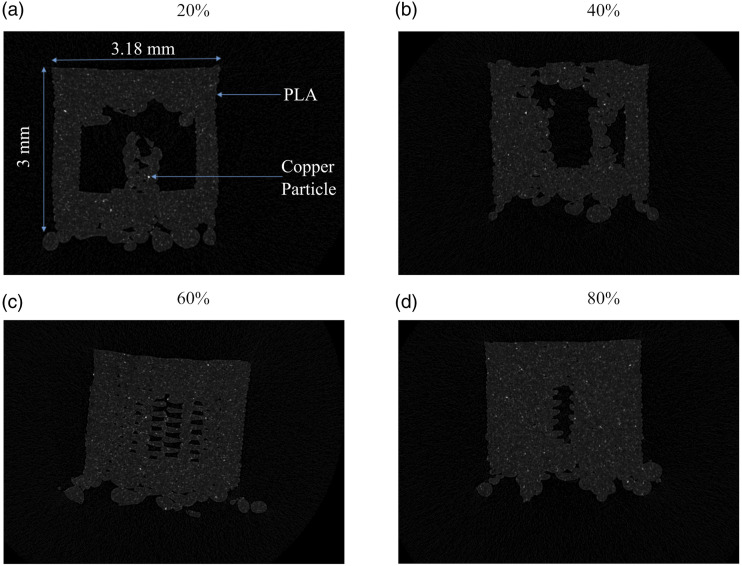
Cross-sectional images using micro-CT of 3D printed dog bone structure with (a) 20%, (b) 40%, (c) 60%, and (d) 80% infill.

After the scans, the reconstruction of Micro-CT data begins. Reconstruction software (Nrecon version 1.7.1.0, Bruker, Belgium) processes and reconstructs the acquired images. Various filters can be applied based on the artifacts to remove them. Ring artifact correction of 4 and beam hardening correction of 50% was used. Figure 4 shows the reconstruction of Micro-CT data from the specimen. The small volume of interest (VOI) region is captured and reconstructed to get the top view slices of VOI. It shows how the slice plane is generated from the specimen, which would help to understand Figure 5. Figure 5 shows a cross-sectional view of specimens of 20, 40, 60 and 80 infill percentages. This allows for investigating specimen characteristics such as air gap, material distribution, and particle distribution. This figure also shows the different raster formations in different infill percentages. Image registration software (DataViewer, Bruker, Belgium) was used to reorient the image boundary line with the axis. The software takes the cross-sectional image stack as input and provides three views of the sample, i.e., Coronal, Sagittal and Transverse plane. It allows reorienting of the image in one plane and automatically reorients in another plane.

### Porosity and particle distribution analysis

**Figure 6.**
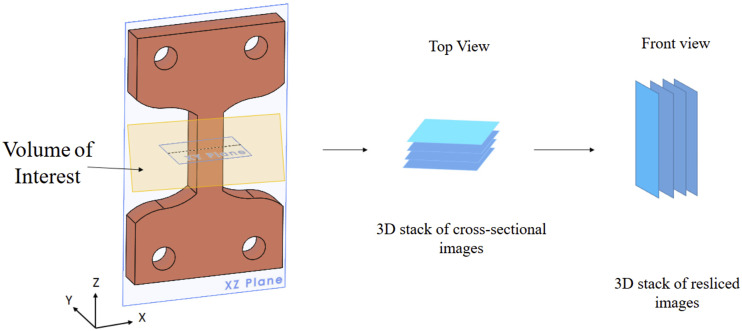
Schematic of a 3D stack of resliced Images. This figure shows the volume of interest of the sample and the resulting image stack of the volume of interest. The dataset was then resliced to provide images in the front plane of the specimen.

**Figure 7.**
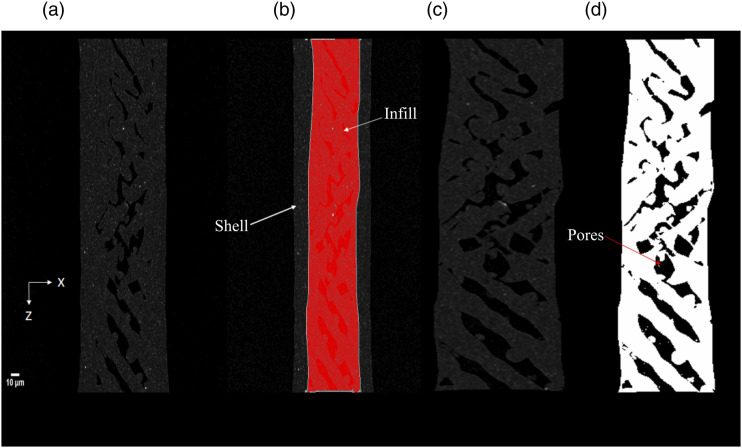
Processing steps for calculating porosity using Micro-CT images (a) Reconstruct the images for top view (b) ROI for infill region whose porosity calculation needs to be done (c) Median filter of three to clear out noise (d) Threshold of six to get the final image with porous region denoted by black.

The acquired images were pre-processed to understand the samples' particle size distribution and porosity. The above results will help in the analysis of the specimen. CTAn (Bruker, Belgium) software was used for all the image pre-processing. For the porosity analysis, the images are reconstructed to obtain the Front View using Dataviewer software, as seen in Figure 6. The front view allows the sample porosity to be investigated in a similar orientation to how the sample was manufactured. The front view makes it easier to process images and further distinguish between the shell and infill region. This shows the process of image reconstruction for further pre-processing analysis. The images are further sliced to get the front view and examine the only raster part for the analysis using software (Dataviewer, Bruker, Belgium), as seen in Figures 6 and 7(a). The image contains both the infill and shell region. The infill portion of the images was examined to obtain the porosity measurement. So, the region of interest is defined manually (denoted in red) to get the infill region, as shown in Figure 7(b). Then a median filter of radius 3 is applied to the region of interest to filter out noise, as seen in Figure 7(c), and then a threshold of 6 is used to obtain the final image, as shown in Figure 7(d). White is the region of material, and black is the porous area. A built-in “3D analysis module” in CTAn was used to find the porosity of the sample.

**Figure 8.**
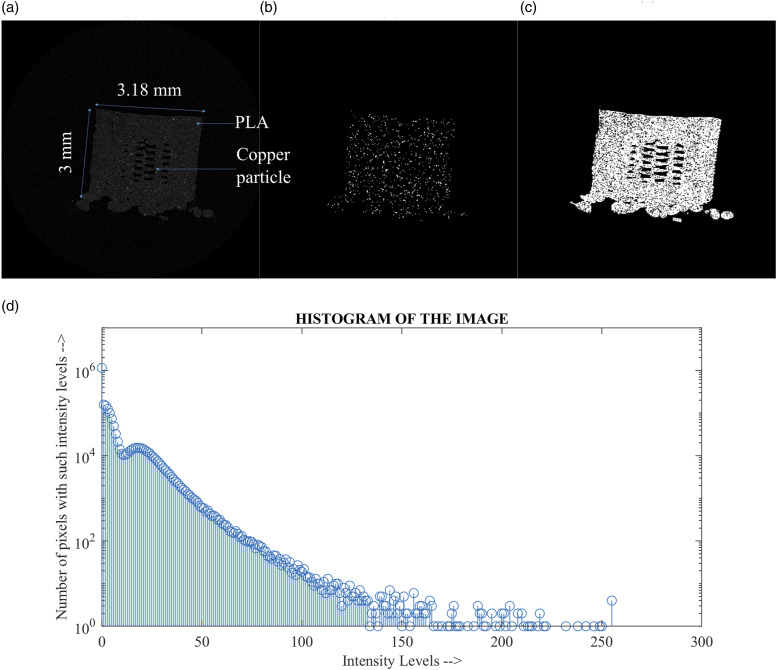
(a)Cross-sectional image 60% infill (b) Copper material distribution using a threshold of 42 (c) Threshold image showing PLA material in white colour (d)Histogram of the cross-sectional image.

Figure 8 demonstrates the image processing steps for the FFF sample. Figure 8(a) shows the original image of the sample, Figure 8(b) shows the identified copper particles, and Figures 3–8(c) shows the PLA matrix material. A histogram was generated for the particle distribution, as shown in Figure 8(d). In general, 0 indicates porous spaces where X-rays did not hit the specimen, and 255 means 100% X-ray beams were either absorbed or deflected. The denser the material is, it is tended more toward 255 since absorption will be greater. The objective is to segment both PLA and copper material to find the sample’s porosity, particle size and count. Since copper has a higher density than PLA material, a threshold of 42 was applied. Therefore, the copper particles are represented as white, as shown in Figure 8(b). Different threshold range helps to provide a distribution of materials within the sample cross-sections. For example, a threshold range of 14 to 42 gave the PLA materials in white, as shown in Figures 3–8(c). The copper particle distribution was found using a built-in 3D particle analyzer.

### DVC analysis of FFF samples

For DVC, a MATLAB-based program (ALDVC 1.0) was used.^24^ The program compares the original and deformed images to find the 3D deformation and strain values of the specimen. A server computer (precision T5600, Dell, Round Rock, Texas) with a RAM of 112 GB and two Intel Xeon CPU E5-2680 2701 Mhz 8-core processors were used for DVC analysis.

Several parameters need to be defined to find volumetric deformation and strains. First is the subset size, a large subset of 128 cubic voxels is initially assumed based on the expected deformation. Then, it is iterated to a lower subset based on the best correlation. After selecting the subset size, different voxel spacing or step sizes are used to find the best correlation. Based on this, a final subset size of 64 cubic voxels and 32 (50%) voxel spacing was utilized. For initial guess estimation, zero normalized cross-correlation is used for local correlation since it is most suitable for small deformations. Then, the finite difference method is used for global correlation, which is the recommended method by the software. The DVC analysis parameters used in this study are summarized in Table 3. The finite difference method is used based on infinitesimal strain for strain measurement.

**Table 3.** Digital volume correlation parameter values.

Parameter	Values
Subset size	64 cubic voxels
Subset spacing	32 voxels
Initial guess estimation	Zero normalized cross-correlation
Strain measurement	Infinitesimal strain

## Results and discussion

### Porosity and particle distribution

**Figure 9.**
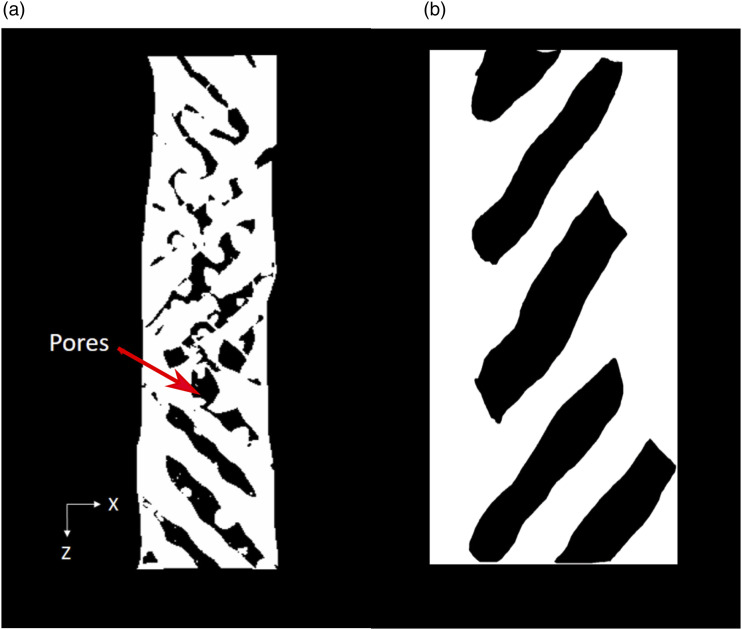
(a) Pore distribution on a resliced plane (b) Pore distribution of microscopic image.

From the pre-processing of the CT images, as shown in Figure 9(a) of 60% infill 3D printed part, it was found that the porosity is 33.97%, less than the expected porosity of 40%. This might be due to various reasons. The tolerance for the Prusa i3 mk2 printer is insufficient for printing the narrow section of the dogbone, which is just 2.38 mm in width. Also, it might be because of printing parameters like the printing speed or the printer’s nozzle size.^25,26^ The current study does not focus on the printing parameters and their optimization. Sofiane *et al.* worked with 100% infill 3D printed Nylon and found significant porosity variation across the samples using Micro-CT data analysis.^27^

To further verify the porosity results, a microscopic image, Figure 9(b), of the 60% infill pattern was captured and processed to calculate porosity. The porosity was found to be 31.69% which is in the range of Micro-CT image results. The main difference is that microscopic images only capture a single layer of 3D printed parts for processing, which might not hold for the whole stack of layers; that is why Micro-CT scanned images are advantageous for internal feature measurements.

**Figure 10.**
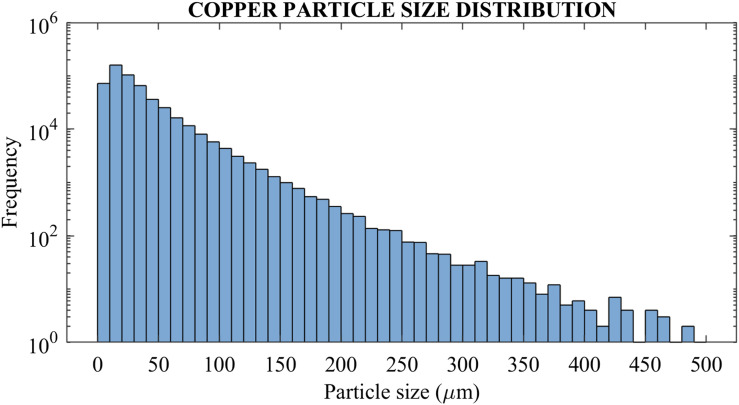
Copper particle distribution for 60% infill specimen.

As seen in Figure 10, a histogram of particle distribution is generated. The figure depicts the frequency of the particle size range. It is visible that copper particle diameter between the range of 10–20 μm has the highest frequency and after that high percentage of copper particle diameter in the range of 20–30 μm. Copper particle size distribution is significant because this particle acts as a correlation marker for the DVC process, which helps detect tiny movements in the loaded parts. The features should be small enough to capture small movements under loading conditions. For the resolution of 5.4 μm, the particles in the 10–30 μm range will be sufficient for markers to detect movements.^28^ Above 100 μm diameter particles are less than 2% of total particles and are believed to be present because of coagulation of small particles after nozzle heating which cannot be resolved using the above image processing techniques and is out of scope for the current research.

### Deformation and strain fields

**Figure 11.**
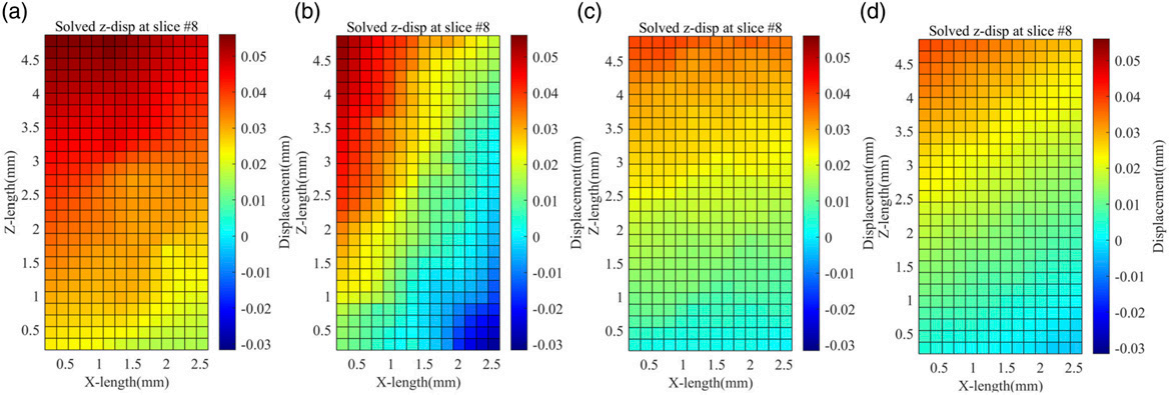
Z-Displacement field across X-Z plane for (a) 20%, (b) 40%, (c) 60%, and (d) 80% infill specimen for 150 N of applied force.

The DVC results provide extensive information on the behaviour using the displacement and strain maps. Figure 11 shows the displacement along the axis of the applied force from 50 N to 150 N for each infill percentage specimen. As the infill percentage increases from 20 to 80 percent, the magnitude of displacement decreases. This can be explained by the fact that the infill percentage increases the stiffness. Hence as stiffness increases, displacement decreases. The displacement from the load cell side decreases towards the other side of the specimen. As can be seen from the images, the left side of the specimen has more displacement compared to the right side, which might be because of a small sample misalignment in the testing stage.

**Figure 12.**
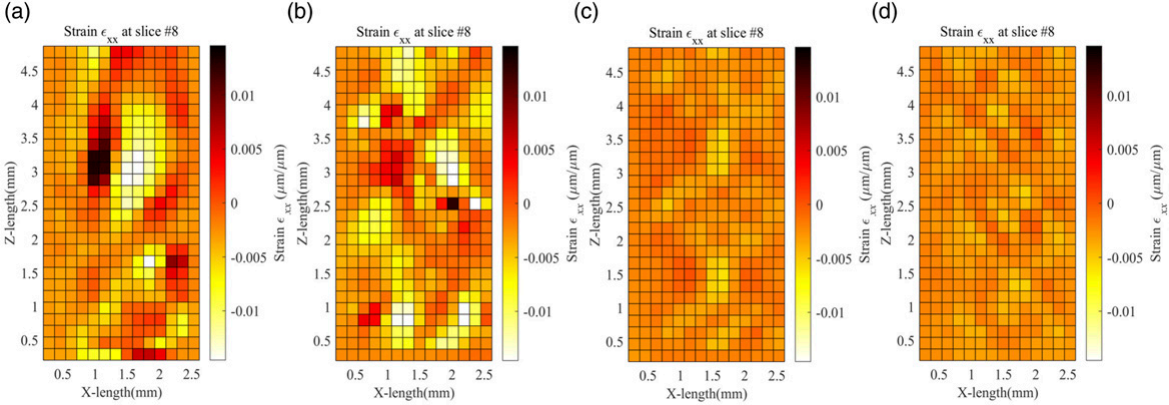
Volumetric Strain $\varepsilon_{xx}$ across XZ plane for (a) 20%, (b) 40%, (c) 60%, and (d) 80% infill.

Similar to displacement, Figure 12 shows the longitudinal strain ( $\varepsilon_{xx}$ ) along the XZ plane for different infill percentages. The strain is negative across all the infill percentages from 20% to 80%, apart from some regions along the raster having a positive strain. This might be because of uneven force distribution along the axis during tensile setup. Additionally, the different infill percentages affect the internal strain field distribution. The orientation of the infill will also influence strain within the test samples. The $\varepsilon_{xx}$ strain decreases with an increase in infill percentage because of the additional material in the cross-section. A uniform negative strain can be seen along the shell region of the specimens.

**Figure 13.**
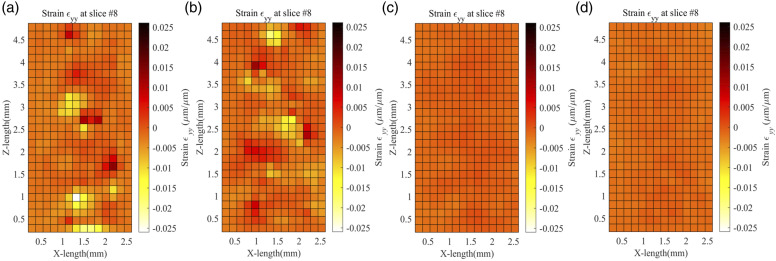
Volumetric Strain $\varepsilon_{yy}$ across XZ plane for (a) 20%, (b) 40%, (c) 60%, and (d) 80% infill.

Figure 13 below shows the volumetric strain $\varepsilon_{yy}$ along the X-Z plane. It has the same behaviour as $\varepsilon_{xx}$ strain. The strain values are negative along the X-Z plane for all infill percentages, with some positive strain values along the raster for similar reasons as mentioned above. Also, the strain decreases with increasing infill percentage. The negative strain values indicate that the sample is contracting along the y-axis. There is also high negative strain across the borders which indicates stress zones. It makes sense as the stress zone is discontinuous because of the raster connecting with the side walls. Timpano *et al.* in their study found $\varepsilon_{xx}$ and $\varepsilon_{yy}$ strain values to be negative, indicating the shrinking of the sample due to extension along the z-axis.^23^

**Figure 14.**
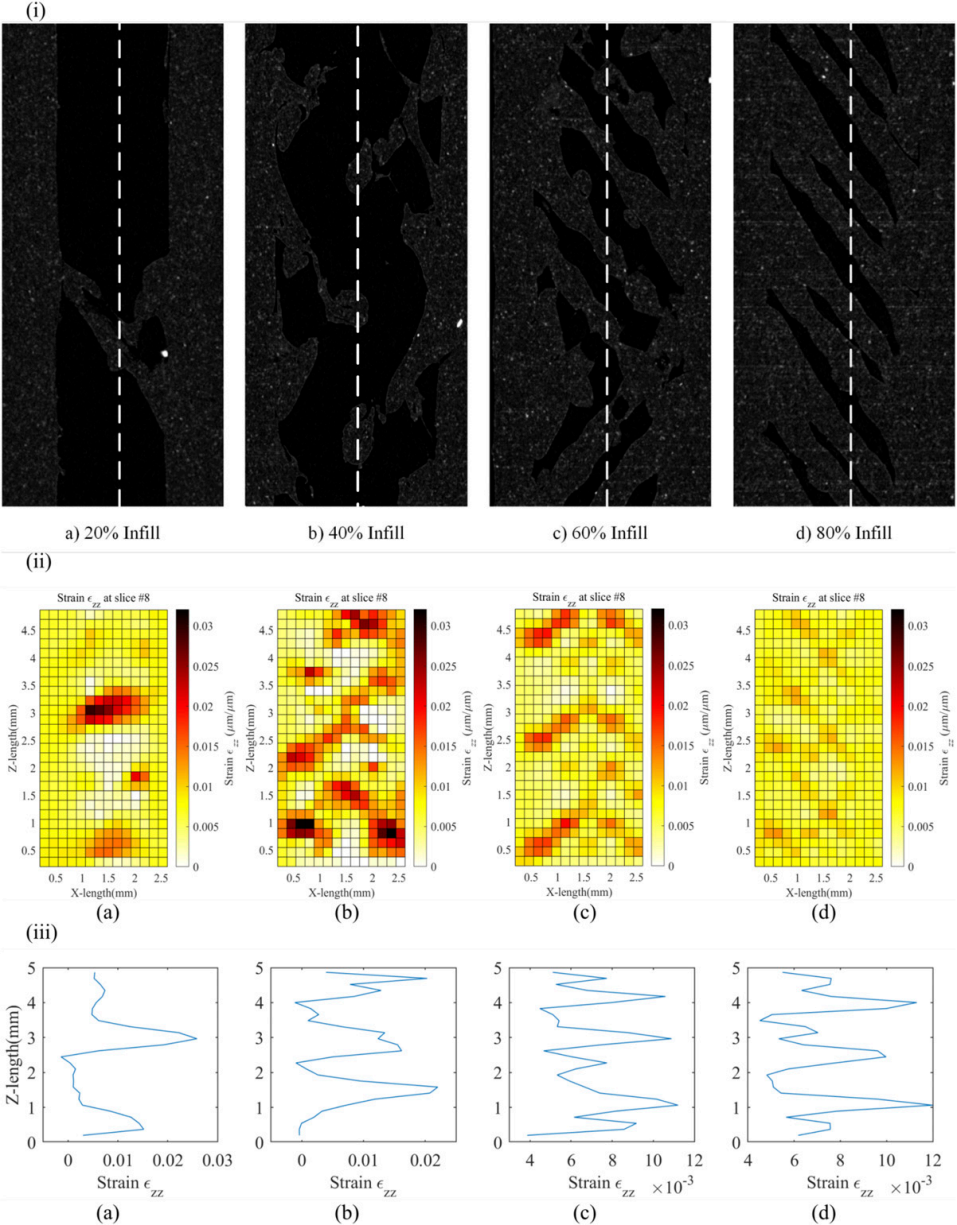
(i) X-Z plane view of reconstructed 150 N MicroCT data (ii) Volumetric Strain $\varepsilon_{zz}$ across XZ plane (iii) Strain $\varepsilon_{zz}$ across the Z-length for (a)20% infill, (b) 40% infill, (c) 60% infill, (d) 80% infill.

Figure 14 compares the Micro-CT image with the strain map and explains how infill distribution affects the resulting strain. Figure 14(i) shows the Micro-CT images obtained for the 20, 40, 60, and 80 infill samples. Figure 14(ii) shows the $\varepsilon_{zz}$ across the different infill percentages i.e., the direction of strain. As the infill percentage increases from 20 to 80, a decrease in strain is observed since there is more material to reinforce the object. It also shows the region of high internal strain. The high strains forms along +45/−45 bands, which correspond with the raster orientation. As can be seen from the figure, the strain bands are smoother as the infill percentage increases, which might be because as the infill percentage decreases, the raster might be less precise for the dogbone structure. Also, there may be gaps or discontinuity along the raster bands as the infill percentage decreases. The 20% infill samples have the greatest porosity and therefore the greatest variation in strain. Conversely, the 80% infill samples have less porosity and therefore strain is more consistent though the cross-section of the sample. Other printing parameters, such as bed temperature gradients and alignment, can affect the 3D printed geometry. The high strains are also visible at the junction of the raster with the shell of the structure. The crack initiation can also be detected with the strain map, which under the current loading condition, is not possible. These maps can be generated for multiple slices across the thickness of the specimen and can help capture strain for anisotropy of the FFF specimen. Figure 14(iii) shows the strain distribution of $\varepsilon_{zz}$ across the Z-length marked by dotted lines in Figure 14(i). The peak strain has an almost periodic behaviour for each infill percentage, the peak strain is associated with the raster band, and the lower strain almost tends to zero are the gaps between the raster bands. Since 20% infill would have significantly fewer raster bands than 80%, the latter has more peaks. The peak strain for 20% infill is around 0.038, and for the 80% is around 0.012, which is a decrease of 316.6%. Timpano *et al.,* in their study of a 100% infill copper-filled PLA sample, found similar +45/−45 bands along the raster orientation but predominantly +45 strain bands for $\varepsilon_{zz}$ strain.^23^ They also had similar cyclic strain peaks across the sample length, but the loading condition was different. They used 100 N preload with step loading of 150 N and 200 N. Wang *et al.* built the specimen using stereolithography and performed a compression test.^22^ This work showed the volumetric deformation and strain fields developed on a porous structure which has unit-cell as hourglass-shaped, which would not be possible using DIC. Hourglass shape is two trapezoid prism with the smaller face of both sides connected to each other. They found a high tensile strain zone at the top/bottom edge of the hourglass and high compressive and shear strain zones on the side walls. Further, identifying damage forms for the structure. This shows how DVC can map displacement and strain for anisotropic structures like FFF.

Table 4 provides the average strain and standard deviation for 20, 40, 60 and 80% infill specimens calculated using Microsoft Excel. The strain values generated from the ALDVC software over the Volume of Interest (VOI) were imported into Microsoft Excel. The VOI of the sample is 2.9 mm × 2.4 mm × 5.1 mm. The average strain and standard deviation in-built formula are used to calculate the average strain over the VOI. The average strain of 20% infill is found to be lower than 40% infill which might be due to high empty space with zero strain value in VOI which reduces the average strain. For 60% and 80% infill, the average strain is almost the same, but the standard deviation for 60% is high, which indicates the peak strain for 60% is greater than 80%. As discussed in *Porosity and Particle distribution*, it was found that the expected porosity of the 60% infill sample was 33.97% compared to the expected infill percentage of 40%. Due to the tolerances of the printer and the narrow gauge section the actual extruded filament in the cross-section differs from what was expected. This results in the variations in average strain in the VOI region of the test samples.

**Table 4.** Average strain value across the VOI region.

Infill percentage	Average strain
20	0.0072 ± 0.0034
40	0.0086 ± 0.0057
60	0.0071 ± 0.0030
80	0.0072 ± 0.0013

The above results are only based on different infill percentages, which can also be used to understand how much infill percentage would be efficient for the loading condition and cost savings. Also, the results can be used to understand the effect of different print parameters volumetrically, like the effect of layer thickness, shell thickness, and temperature. This would also help in understanding the internal behaviour of complex 3D printed structures, which is impossible with the DIC measurement technique.

Gonabadi *et al.* studied the effect of process parameters like infill pattern and infill density for the PLA samples using DIC.^18^ The results showed full-field surface strain fields; however, meaningful data could not be extracted from the strain maps. The strain distribution showed some strain localizations, which are not correlated to the micromechanical properties of each process parameter. Similarly, Saleh *et al.* used DIC in the study for the mechanical characterization of 3D printed rigid and flexible continuous wire polymer composites.^29^ A full-field internal strain field would have given a more comprehensive analysis of the sample characteristics than a full-field surface strain. Also, the Micro-CT model of the sample could be used to find the volume fraction of the constituents with better accuracy as compared to optical microscopy.

## Conclusions

Tensile tests were conducted on 20, 40, 60 and 80 infill percentage FFF copper-filled PLA specimens while the samples were positioned within a Micro-CT scanner. The specimen was manufactured according to the modified ASTM D638-14 type V. The specimen was scanned in Micro-CT under a preload of 50 N and step load of 150 N. The data was further processed and analysed to find porosity and copper particle distribution in the sample. The ALDVC software performed DVC analysis on 20, 40, 60, and 80 infill percentage samples to measure the internal displacement and strain fields. This work established that displacement increases as the infill percentage decreases due to less material within the sample cross-section. For the volumetric strain $\varepsilon_{zz}$ , a high strain pattern was observed along the +45/−45 raster orientation. Thus, it can be used for understanding the complex internal behaviour of FFF specimens. Furthermore, the data can be used to validate the finite element analysis and analytical models for FFF specimens. Also, the effect of different reinforcement material types and shapes can be explored using the DVC methodology presented in this work and its effect on stress and strain development.
